# Supplementary material for: An On-Demand Dissoluble Chitosan Hydrogel Containing Dynamic Diselenide Bond
Source: Gels. 2021 Feb 20;7(1):21. doi: 10.3390/gels7010021 (PMC7931019; doi:10.3390/gels7010021)
Supplement: Supplementary file 1 [file gels-07-00021-s001.pdf]

Supporting Information for

An On-Demand Dissolution Chitosan Hydrogel Containing Dynamic Diselenide Bond

Xingxia Xu[a], Weihong Lu[a,b*], Jian Zhu[a*], Xiangqiang Pan[a*] and Xiulin Zhu[a]

[a]State and Local Joint Engineering Laboratory for Novel Functional Polymeric Materials, Jiangsu Key Laboratory of Advanced Functional Polymer Design and Application, Department of Polymer Science and Engineering, College of Chemistry, Chemical Engineering and Materials Science, Soochow University, Suzhou 215123, China.

[b]Jiangsu Key Laboratory of Advanced Functional Polymer Design and Application, Department of Polymer Science and Engineering, College of Chemistry, Chemical Engineering and Materials Science and State Key Laboratory of Radiation Medicine and Radiation Protection, School for Radiological and Interdisciplinary Sciences (RAD-X), Soochow University, Suzhou 215123, People's Republic of China

Corresponding author:

Xiangqiang Pan, E-mail: panxq@suda.edu.cn

Jian Zhu, E-mail: chemzhujian@suda.edu.cn.

Weihong Lu, E-mail: luweihong@suda.edu.cn

^{1}H NMR spectra were recorded in D_2O on a Bruker Avance 300 at 300 MHz. Thermogravimetric analysis (TGA) were performed on thermogravimetric analyzer (PerkinElmer, Pyris 1 TGA) at a heating rate of 10 °C min^{-1} from 30 °C to 800 °C under a N_2 atmosphere.

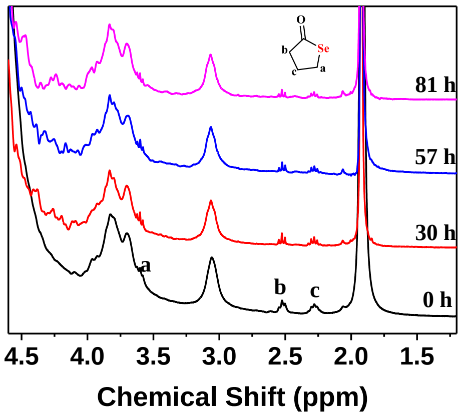

Fig. S1. ^{1}H NMR spectra of the reaction mixture of chitosan and γ-butyroselenolactone (4 mol%) at different intervals.

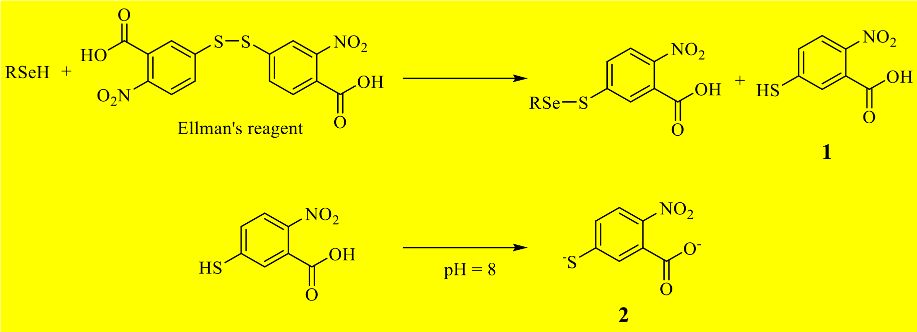

Scheme S1 the reaction of Ellman's reagent with selenol

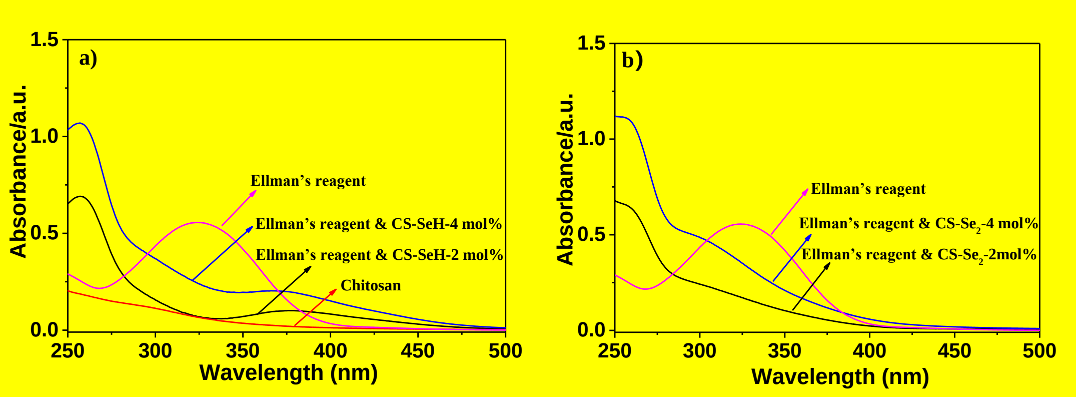

Fig. S2. UV-vis spectra of chitosan, CS-SeH, CS-Se$_2$ after treatment with Ellman's reagent.

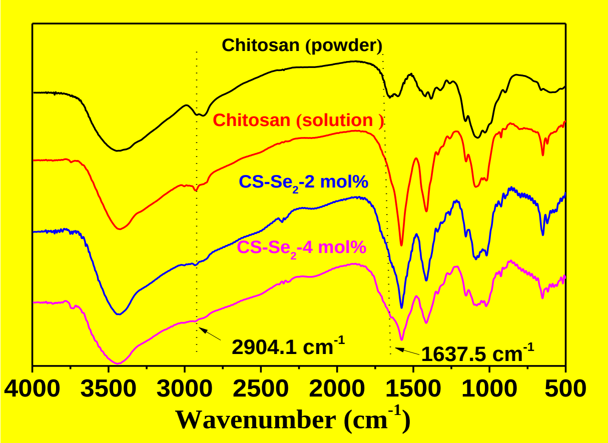

Fig. S3 FT-IR spectra of chitosan, 2 and 4 mol% Se-containing chitosan hydrogels.

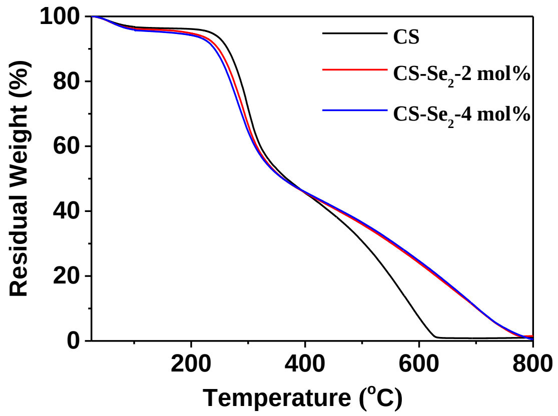

Fig. S4. The TGA curves of chitosan, 2 and 4 mol% Se-containing chitosan hydrogels.
